# Impacts of morphology parameters on the risk of rupture in intracranial aneurysms: statistical and computational analyses

**DOI:** 10.1038/s41598-023-46211-y

**Published:** 2023-11-03

**Authors:** Yujing Wang, Jing Jin, Jie Chen, Peng Chen, Seyyed Amirreza Abdollahi

**Affiliations:** 1 College of Health Informatics, Chongqing Medical University, No.1 Medial Road, 400010 Chongqing, China; 2 Department of Neurosurgery, Chongqing University Cancer Hospital, 400000 Chongqing, China; 3 Faculty of Mechanical Engineering, University of Tabriz, Tabriz, Iran

**Keywords:** Biomedical engineering, Mechanical engineering

## Abstract

The hemodynamic analysis of the blood stream inside the cerebral aneurysms reveals the risk of the aneurysm rupture. In addition, the high risk region prone to rupture would be determined by the hemodynamic analysis of the blood. In present article, computational fluid dynamic is used for the investigation of the hemodynamic effects on the aneurysm wall and risk of rupture. This study tries to find the connection between the risk of rupture with three geometrical features of aneurysm i.e., Ellipsoid Max semi-axis, Size ratio and Tortuosity. Statistical analysis is done over 30 different ruptured /unruptured ICA aneurysms to find meaningful relation between selected geometrical factors and rupture risk. The hemodynamic analysis is done over four distinct aneurysm models to attain more details on effects of chosen geometrical factors. The results of simulations indicate that the Ellipsoid Max semi-axis have meaningful impacts on the risk of rupture.

## Introduction

Aneurysms are abnormal bulges or weak spots that occur in blood vessels, particularly in the arteries of the brain. They pose a significant health risk as they can potentially rupture, leading to life-threatening hemorrhages. The geometrical features of an aneurysm, such as its shape, size, and morphology, play a crucial role in determining the hemodynamic characteristics and the risk of rupture^1–3^.

Understanding the relationship between aneurysm geometrical features and hemodynamic characteristics has become a subject of intense research in the field of vascular biomechanics^4,5^. Two key hemodynamic parameters that have been widely investigated are wall shear stress (WSS) and oscillatory shear index (OSI). WSS refers to the frictional force exerted by flowing blood on the vessel wall, while OSI quantifies the temporal variation in the direction of shear stress^6–8^.

The hemodynamic characteristics of an aneurysm, particularly the WSS and OSI, are influenced by various geometrical features^9,10^. The size of the aneurysm, including its diameter and volume, has a direct impact on the flow patterns and the magnitude of WSS. Larger aneurysms tend to exhibit lower WSS values, which can result in reduced mechanical stimuli on the vessel wall and potentially contribute to an increased risk of rupture^11–13^.

Beyond size, the shape of an aneurysm is also a critical geometrical feature that affects its hemodynamic characteristics. Aneurysms can exhibit different shapes, such as saccular (bulging out on one side) or fusiform (uniform dilation along the vessel)^14–16^. Saccular aneurysms, with their asymmetrical bulges, often experience complex and disturbed flow patterns, leading to regions of low WSS and high OSI. These flow characteristics are associated with increased vulnerability to rupture compared to fusiform aneurysms, which tend to have more uniform flow patterns and higher WSS^17–19^.

In addition to size and shape, other geometric factors, such as aneurysm neck geometry, aspect ratio (aneurysm height-to-neck width ratio), and irregularities in the vessel wall, can also impact the hemodynamic characteristics and rupture risk^20,21^. Aneurysm neck geometry influences the flow dynamics within the aneurysm sac and the local WSS distribution. Higher aspect ratios are often associated with increased rupture risk, as they create conditions for flow impingement and adverse hemodynamic forces.

Advances in medical imaging techniques, such as computed tomography angiography (CTA) and magnetic resonance angiography (MRA), have enabled clinicians and researchers to accurately assess aneurysm geometrical features and study their relationship with hemodynamic characteristics^22,23^. Computational fluid dynamics (CFD) simulations have emerged as valuable tools to model blood flow within aneurysms and quantify the WSS and OSI distribution^24–26^.

Understanding the interplay between aneurysm geometrical features, hemodynamic characteristics, and the risk of rupture is essential for clinical decision-making. It can aid in patient-specific risk assessment, treatment planning, and the development of improved rupture prediction models. This knowledge can guide the selection of appropriate treatment strategies, such as surgical clipping or endovascular coiling, based on aneurysm characteristics and the associated hemodynamic risks.

In this paper, we aim to investigate the impact of aneurysm geometrical features on hemodynamic characteristics, specifically WSS and OSI, and their correlation with the risk of rupture. By analyzing patient-specific geometries and performing CFD simulations, we seek to deepen our understanding of the complex relationship between aneurysm morphology, hemodynamics, and rupture potential. The findings of this study may contribute to the development of improved diagnostic and therapeutic strategies for managing cerebral aneurysms, ultimately enhancing patient outcomes and reducing the risk of rupture-associated morbidity and mortality.

The aim of this study is to find the meaningful connection between the geometrical characteristics of the aneurysms and aneurysm rupture risk. This study applied statistical method to define the connection between the three important geometrical features of aneurysms and rupture risk over 30 ICA real cases. Besides, hemodynamic analysis is done to reveal more details about the effects on blood flow characteristics on induced WSS, OSI and pressure on aneurysm wall under influence of these geometrical aspects.

## Computational and statistical methods

### Aneurysm selection

It is confirming that all methods were carried out in accordance with relevant guidelines and regulations. Besides, all experimental protocols were approved by of the Ca' Granda Niguarda Hospital and it is confirmed that informed consent was obtained from all subjects and/or their legal guardian(s). All study are approved by Ca' Granda Niguarda Hospital ethics committee.

In the first step, statistical student t-test is performed on geometrical feature of 30 different ICA aneurysms attained from Aneurisk^26^. Among these selected aneurysms, there are 9 ruptured aneurysms while others are un-ruptured. Since the number of the samples are not high enough, results of student t-tests are acceptable when its probable distribution is normal in both ruptured and unruptred aneurysms. Hence, Normality test (Anderson–Darling) is done over these two groups for different geometrical features. It is found that there are three unrelated factors (Size Ratio, Tortusity and ellipsoid max semi axis) which have normal distributions in the selected samples. Then, results of student t-test indicate that average of ellipsoid max semi axis in ruptured samples is meaningfully less than those in unruptured ones. However, the evaluation of the two other geometrical features of Size Ratio and Tortusity on the chosen ICA aneurysm samples show that there is no meaningful direct connection on rupture risk of ICA aneurysms. Nevertheless, these two factors have direct relations on both average velocity of the incoming blood into the sac region and average wall shear stress on sac wall^27–29^.

**Figure 1:**
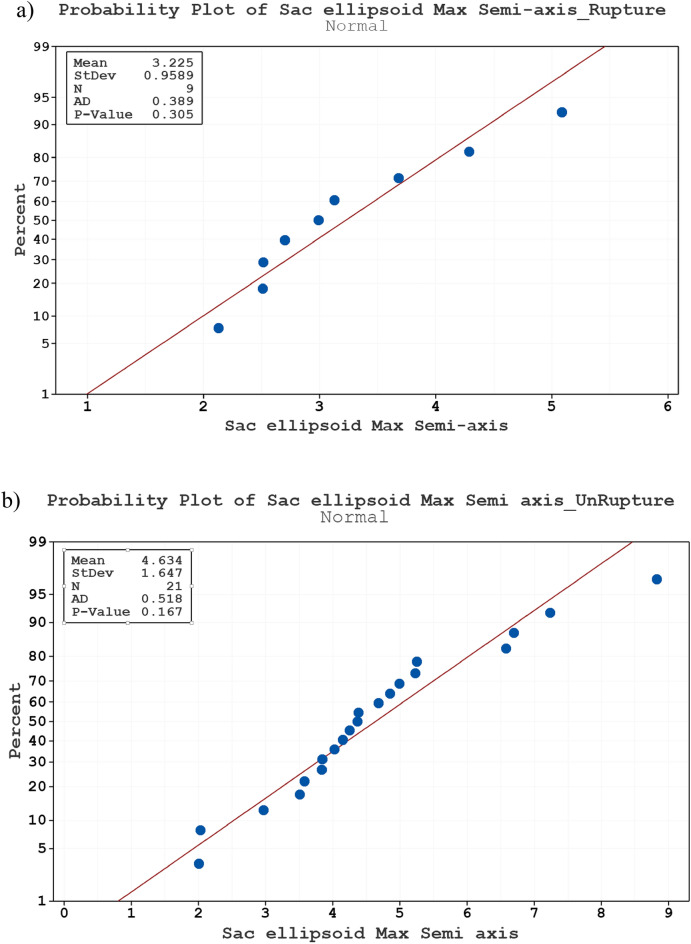
Normality test (Anderson–Darling) for Sac ellipsoid max semi-axis of (**a**) ruptured (**b**) unruptured aneurysms.

Figure 1a,b display Normality test (Anderson–Darling) for Sac ellipsoid max semi-axis of ruptured and unruptured aneurysms, respectively. The P-value of both plots confirms that these data have normal distribution and meaningful effects on the ruptured of aneurysms. The average of ellipsoid max semi-axis on unruptured and ruptured cases is 3.225 and 4.634, respectively.

**Figure 2:**
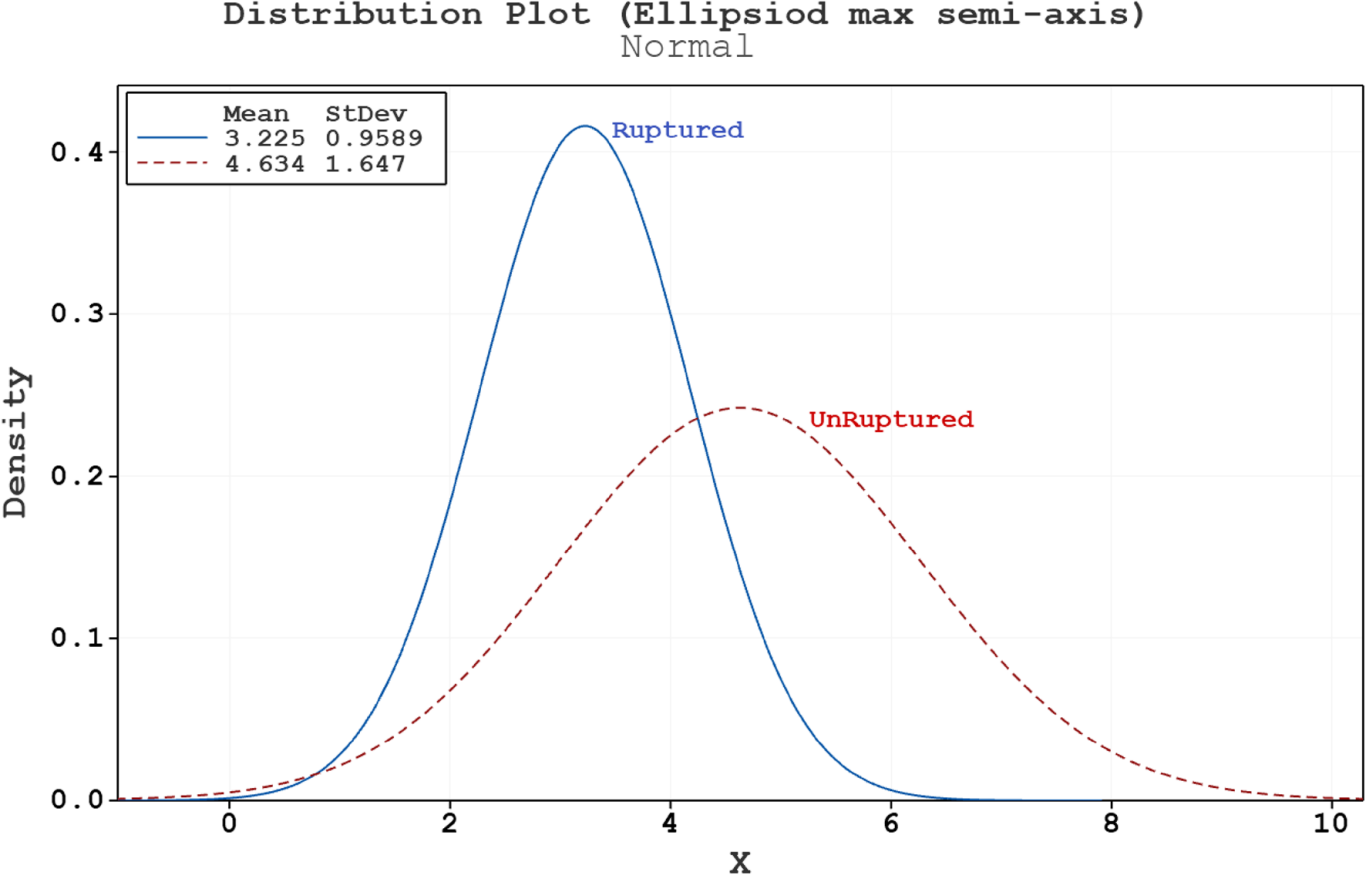
Normal distribution plot for Sac ellipsoid max semi-axis of ruptured and unruptured aneurysms.

Figure 2 demonstrates the results of the student T-test for both samples group and it is observed that the p-value of these data is 0.004 which is less than 0.05 and confirm the connection between ruptured aneurysms and this factor. Hence, in the present work, this factor is chosen for the evaluation of hemodynamic factors i.e., WSS, pressure and OSI on the sac surface via computational simulations.

**Figure 3:**
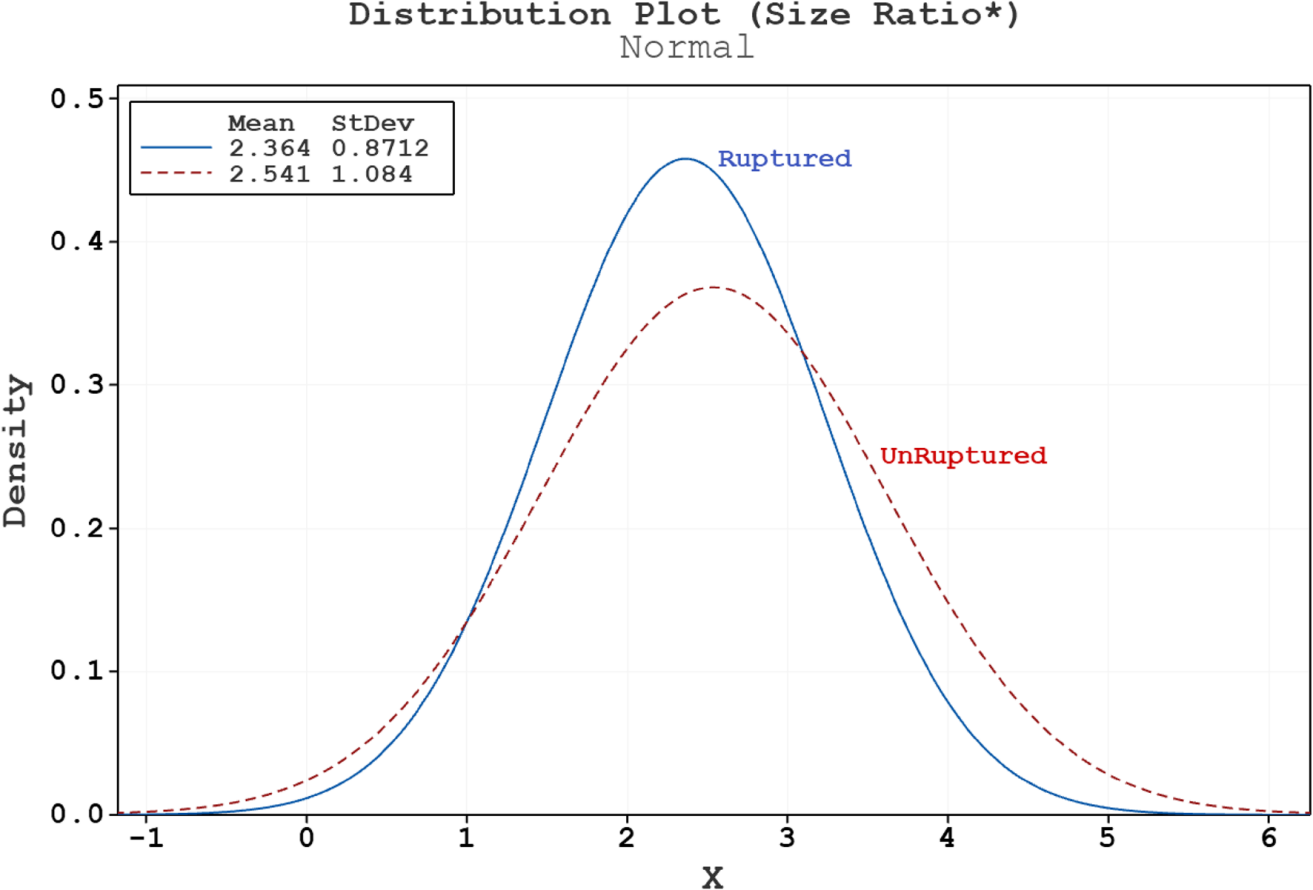
Normal distribution plot for size ratio* of ruptured and unruptured aneurysms.

**Figure 4:**
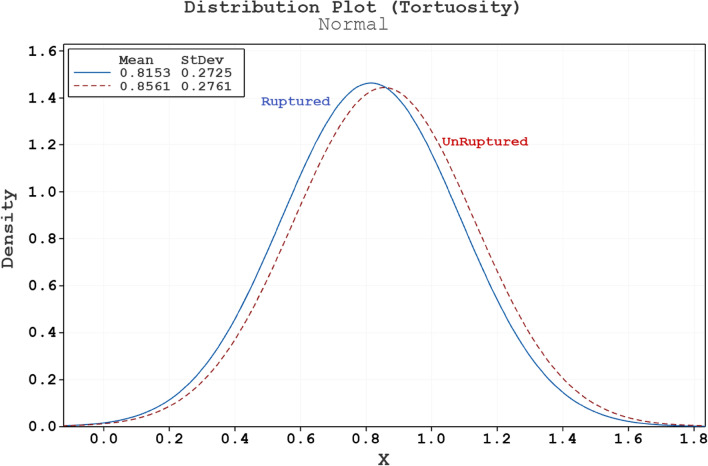
Normal distribution plot for tortuosity of ruptured and unruptured aneurysms.

Figures 3 and 4 demonstrate the results of Normality test of size ratio* and tortuosity on both ruptured and unruptured samples, respectively. As presented in Table 1, the p-value of these two factor is higher than 0.05 and there is no meaningful connection between these two factors and rupture risk.

**Table 1:** Student t-test results of ruptured and unruptured cases.

Parameter	P-value	Threshold	Student t-test result
Max semi-axis	0.004	< 0.05	Significant difference
Size ratio*	0.321	> 0.05	Non-significant difference
Tortuosity	0.357	> 0.05	Non-significant difference

### Geometric feature of selected Aneurysm

**Figure 5:**
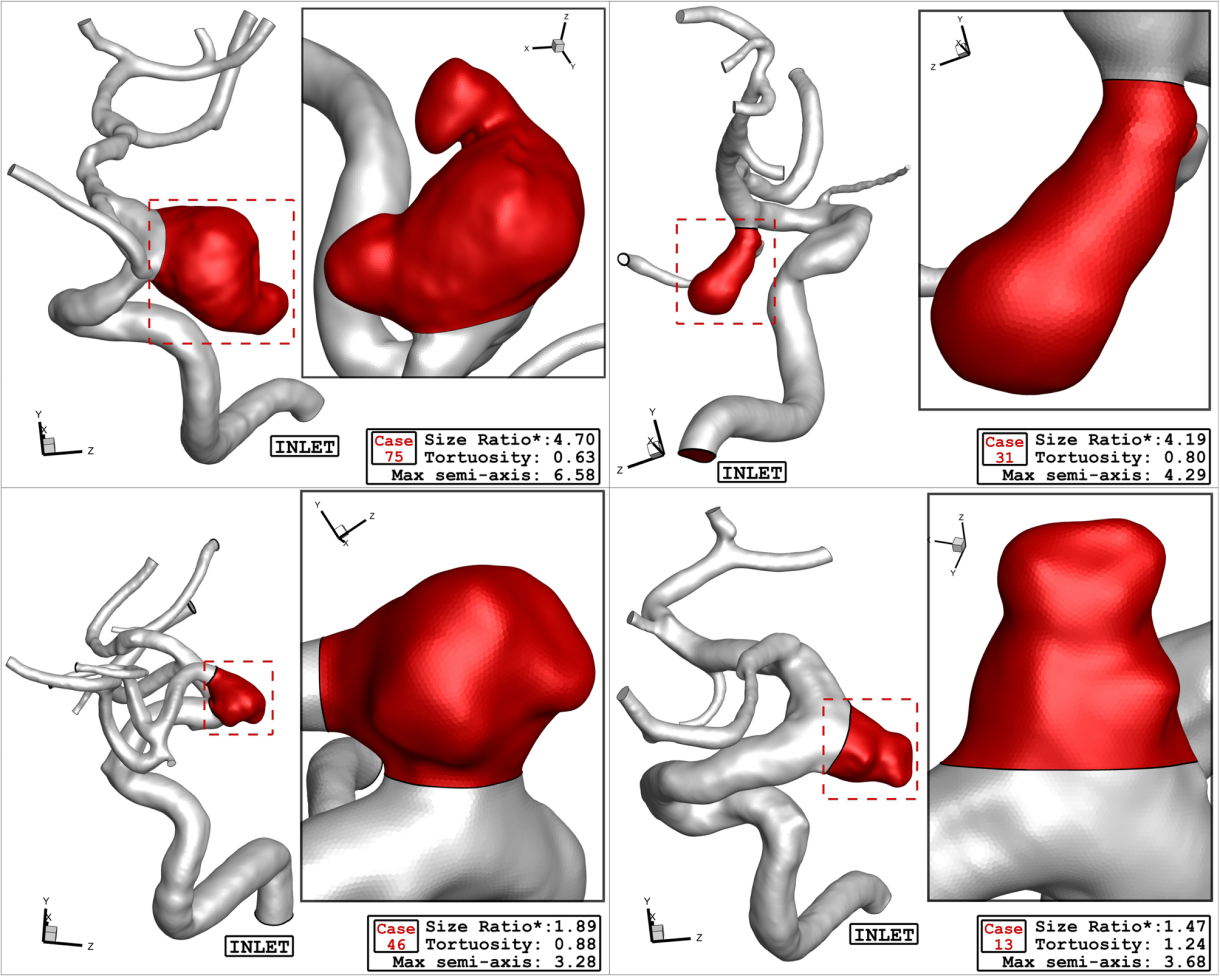
ICA Aneurysm of four different cases.

**Figure 6:**
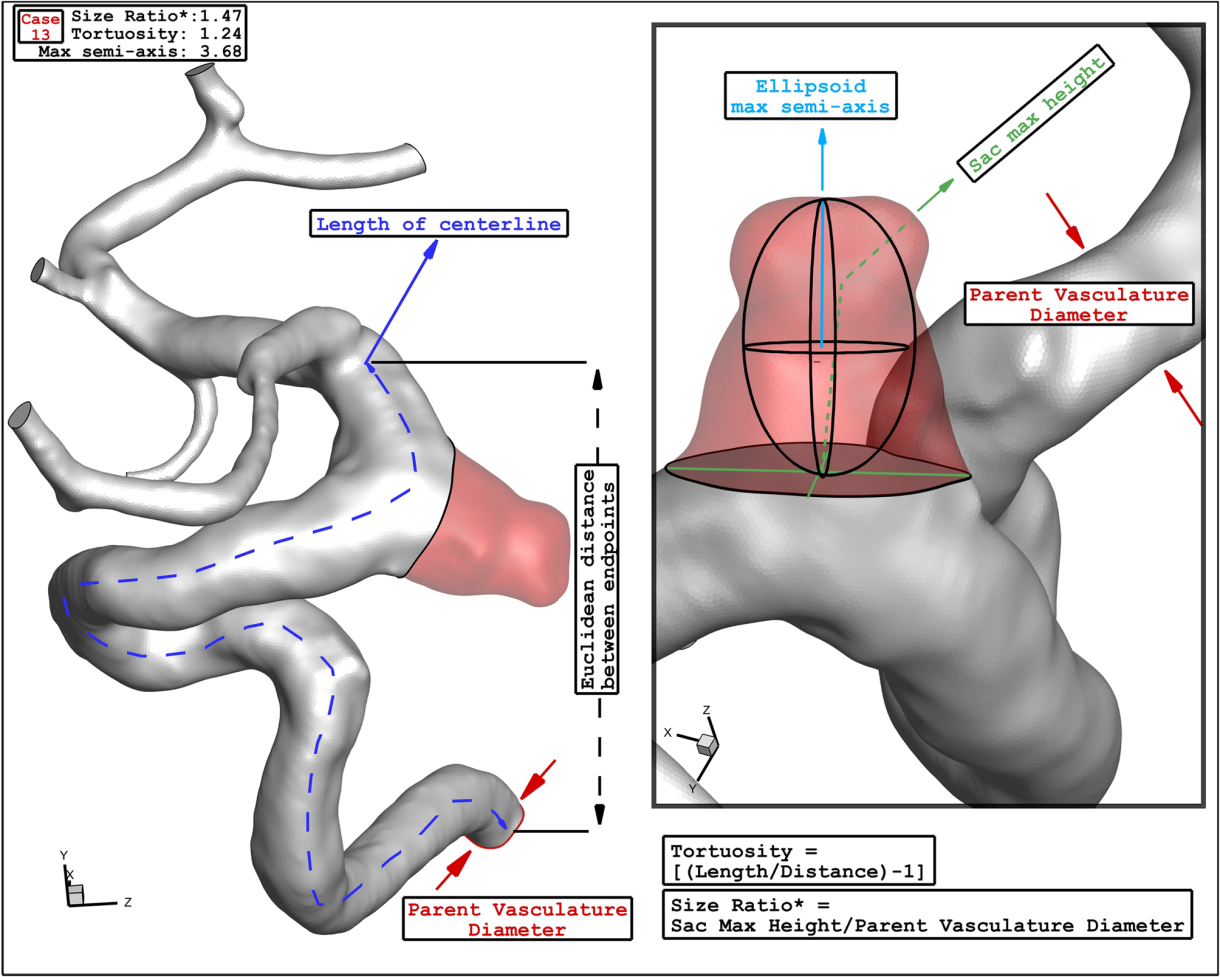
Definition of geometrical parameters.

In this study, four specific geometries of ICA aneurysms with different values of Size Ratio, Tortusity and ellipsoid max semi axis (Fig. 5). Geometrical details of these four samples are presented in Table 2. The definitions of these factors are also displayed in Fig. 6.

**Table 2:** Geometrical parameters of selected cases.

Case no	Size ratio*	Tortuosity	Ellipsoid max semi-axis	Sex	Rupture status	Aneurysm type
75	4.70	0.63	6.58	Female	Unruptured	Lateral
31	4.19	0.80	4.29	Female	Ruptured	Lateral
46	1.89	0.88	3.28	Male	Unruptured	Terminal
13	1.47	1.24	3.68	Female	Ruptured	Lateral

## Governing equations and applied numerical technique

**Figure 7:**
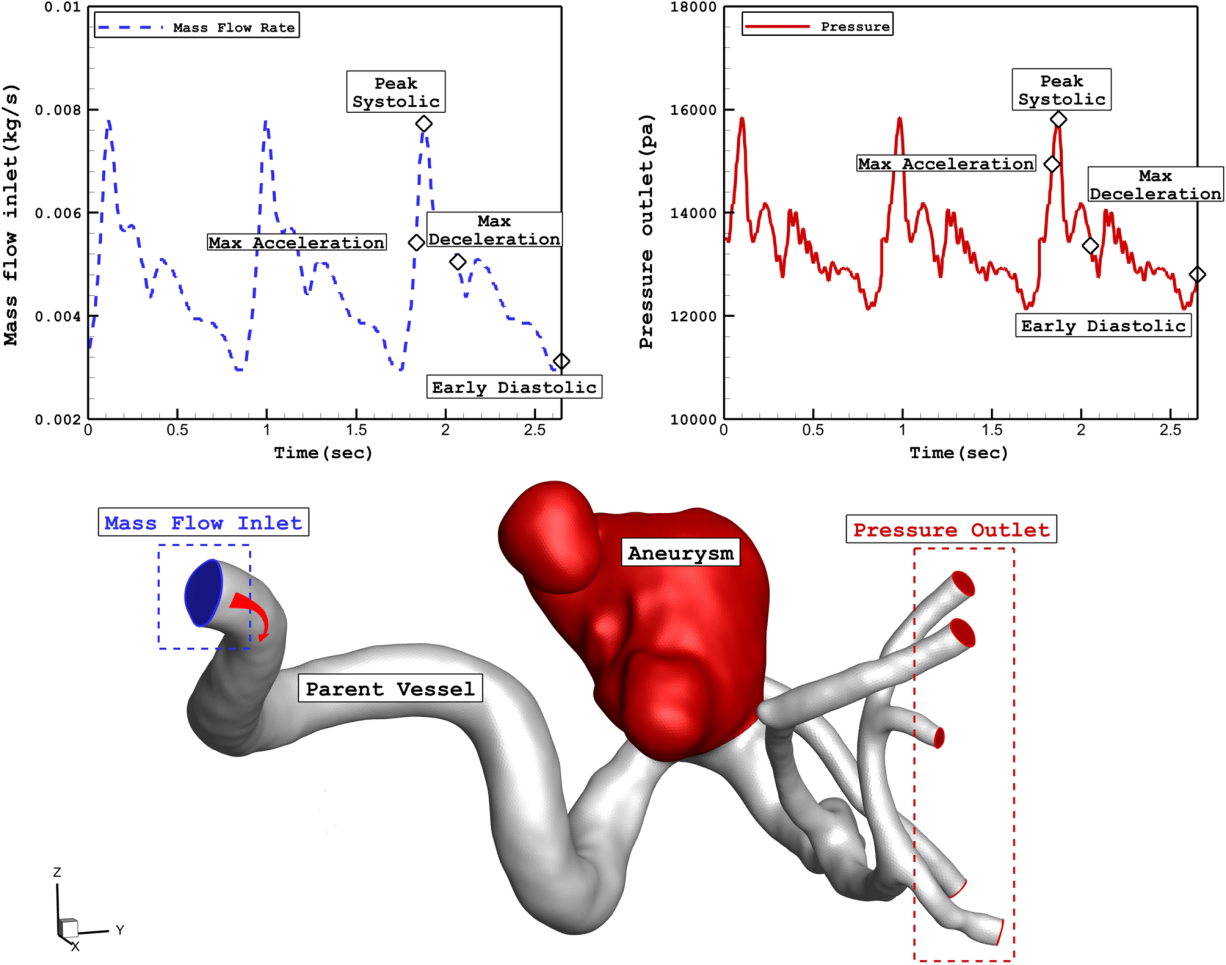
Applied mass and pressure profile at inlet and outlets.

The simulation of the blood stream inside the cerebral aneurysm is performed using transient, incompressible Navier­stokes equations and one-way FSI is applied to model the interaction of the aneurysm wall with blood stream^30,31^. Casson model is applied to calculate the viscosity of the blood in which hematocrit value is defined in this correlation^32^. Applied boundary conditions of the selected model are demonstrated in Fig. 7. The profile of three cardiac cycle on inlet and outlet is also presented in the figure. At inlet, mass flow rate is applied while outlet pressure is applied on outlet. Four specific stages on the applied profile are defined for the hemodynamic analysis. ANSYS-FLUENT software^33^ is used for the simulation of the blood flow inside the aneurysms^34–37^. The computer simulation is widely applied for the simulations biomedical systems^38–42^

**Figure 8:**
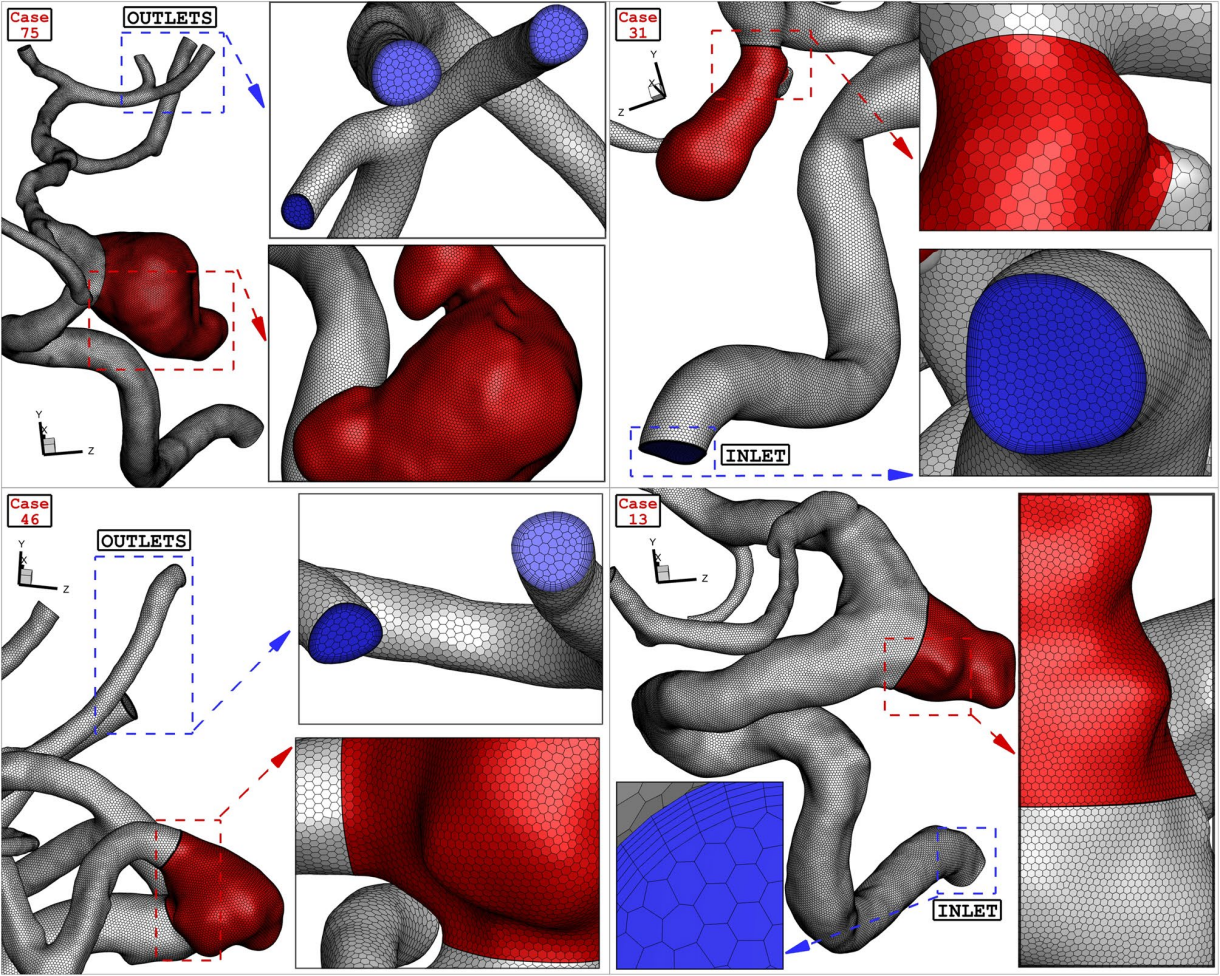
Grid generation of four different cases.

The grids produced for the chosen ICA aneurysms are demonstrated in Fig. 8. Hexagonal structured grids are applied for the chosen aneurysm and parent vessel. The size of produced grid near the aneurysm wall and sac region is lower than other regions. The average number of grid cells for these aneurysms is 2,400,000 cells. Efficient computational data is attained via computational approach^43–47^ when grid size is adjusted based on the importance of the flow^48–51^.

## Results and discussion

**Figure 9:**
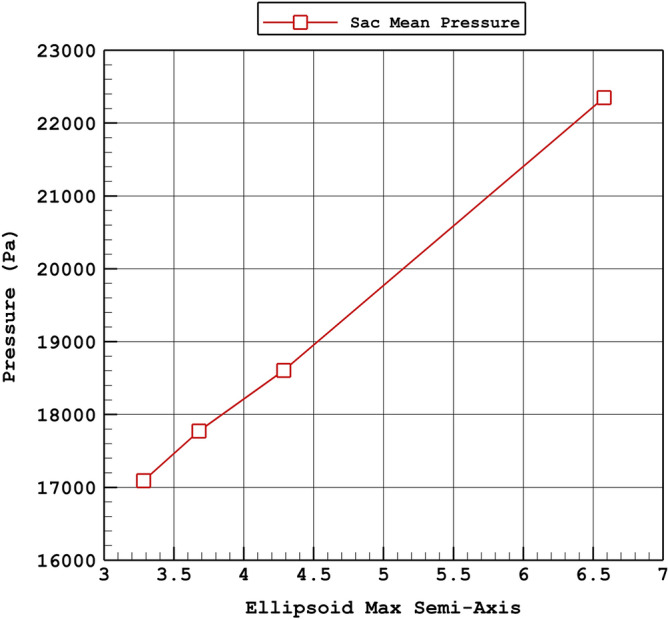
Variation of aneurysm wall mean pressure Vs. max semi-axis (peak systolic).

**Figure 10:**
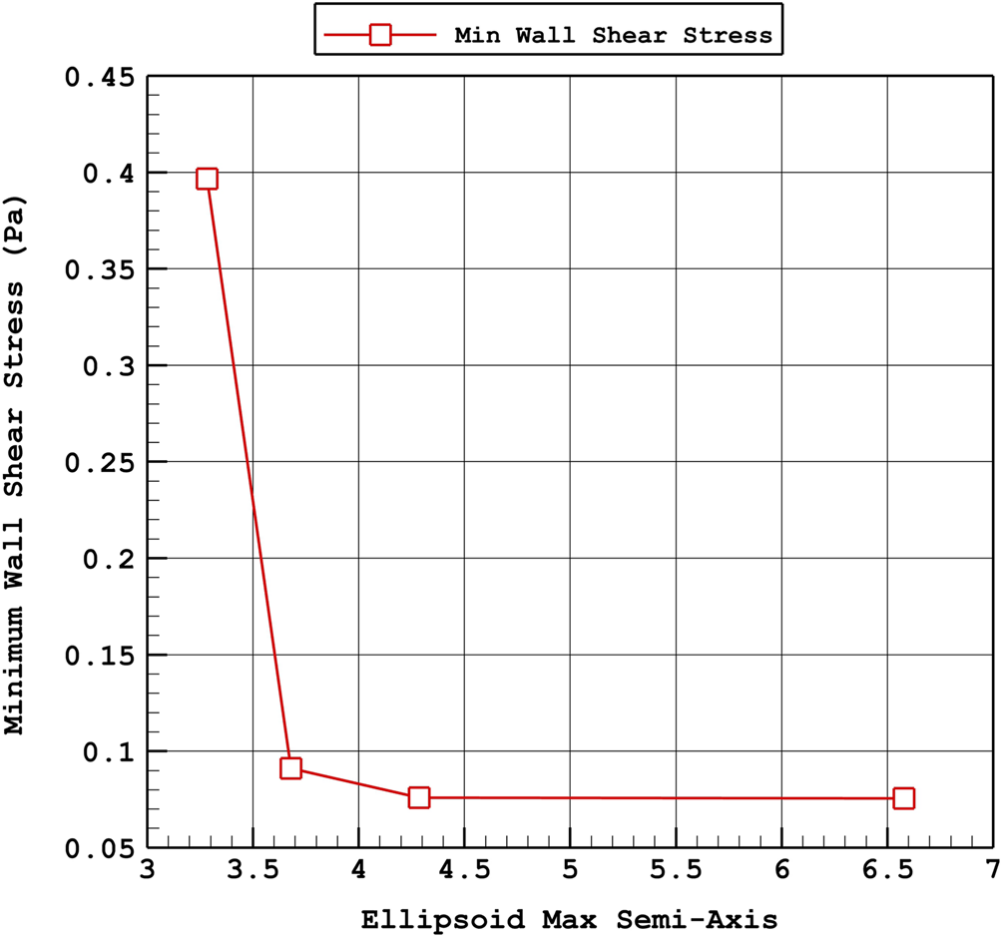
Variation of aneurysm wall minimum WSS Vs. max semi-axis (peak systolic).

The variations of the sac mean pressure and minimum wall shear stress in diverse values of the Ellipsoid Max Semi-Axis are demonstrated in Fig. 9 and 10, respectively. The changes of the Ellipsoid Max Semi-Axis directly indicate that raise of Ellipsoid Max Semi-Axis directly effects on the sac mean pressure. The variation of the Minimum wall shear stress which is important for the rupture of the aneurysm also confirm the importance of the Ellipsoid Max Semi-Axis. In fact, increasing the Ellipsoid Max Semi-Axis would decrease the minimum wall shear stress for the chosen aneurysms.

**Figure 11:**
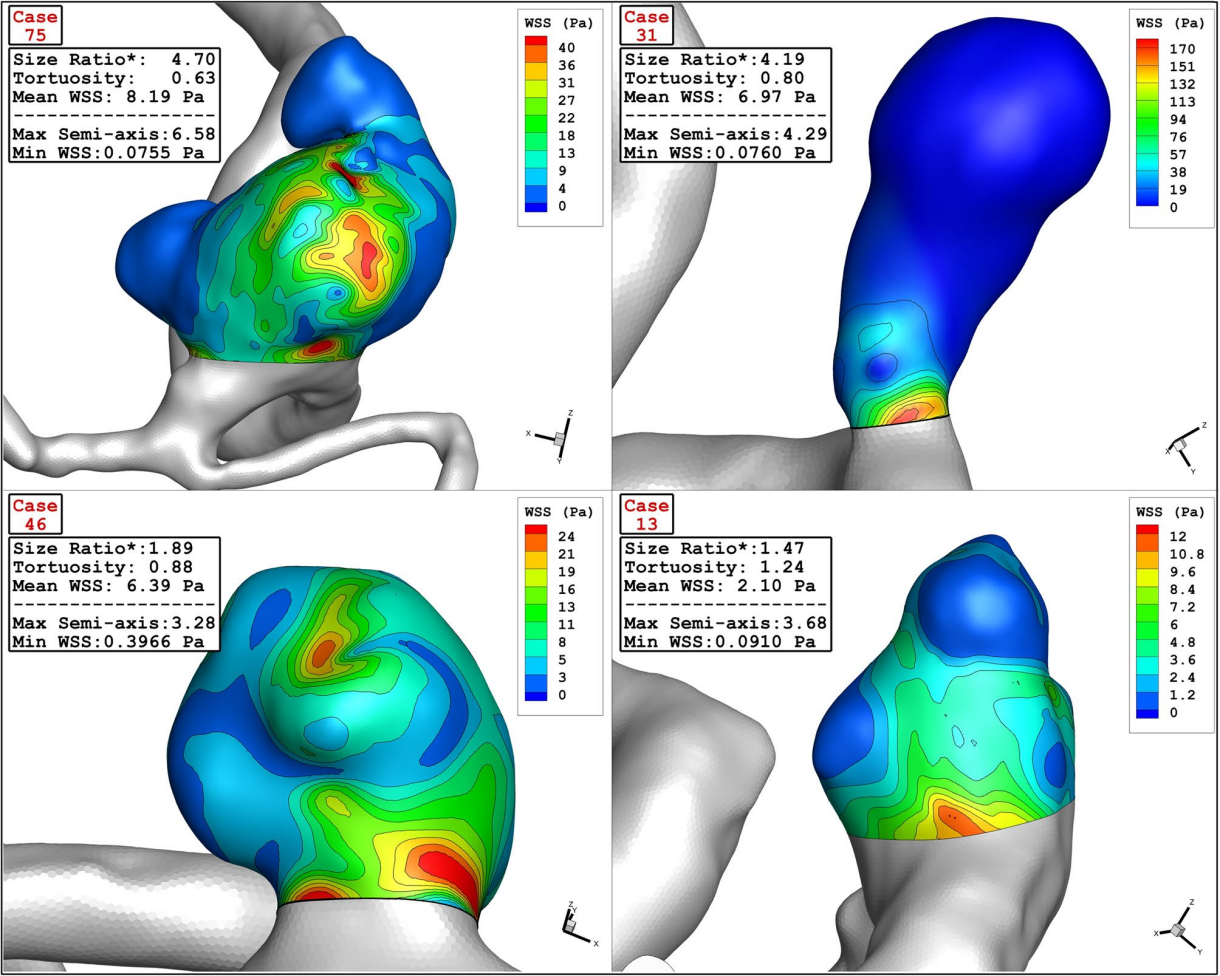
Wall Shear Stress contour of four selected cases (peak systolic).

**Figure 12:**
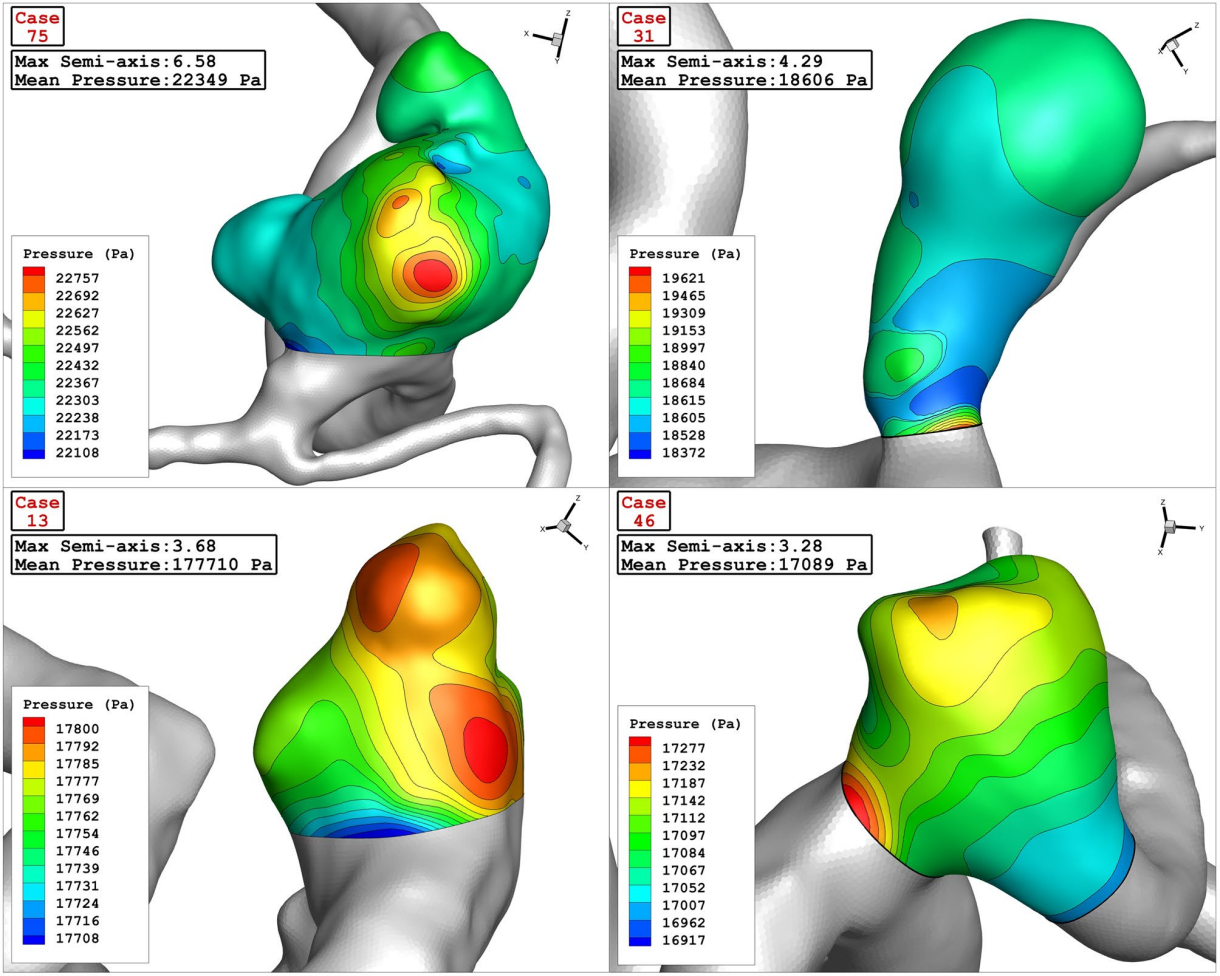
Pressure contour of four selected cases (Max semi-axis effect)—(peak systolic).

The Contour of wall shear stress on the selected aneurysms at peak systolic stage is displayed in Fig. 11. In high Max semi-Axis (Case 75), the critical region, where the value of WSS is high, occurs in dome of the aneurysm sac while critical region of other cases happens near the neck region. Figure 12 illustrates the contour of pressure on sac surface at peak systolic stage. Unlike WSS, the high pressure region is observed on the dome of the aneurysm. It is also noticed that the pressure raises in the region with sharp curvature. The value of mean pressure is also presented in this figure for each cases.

**Figure 13:**
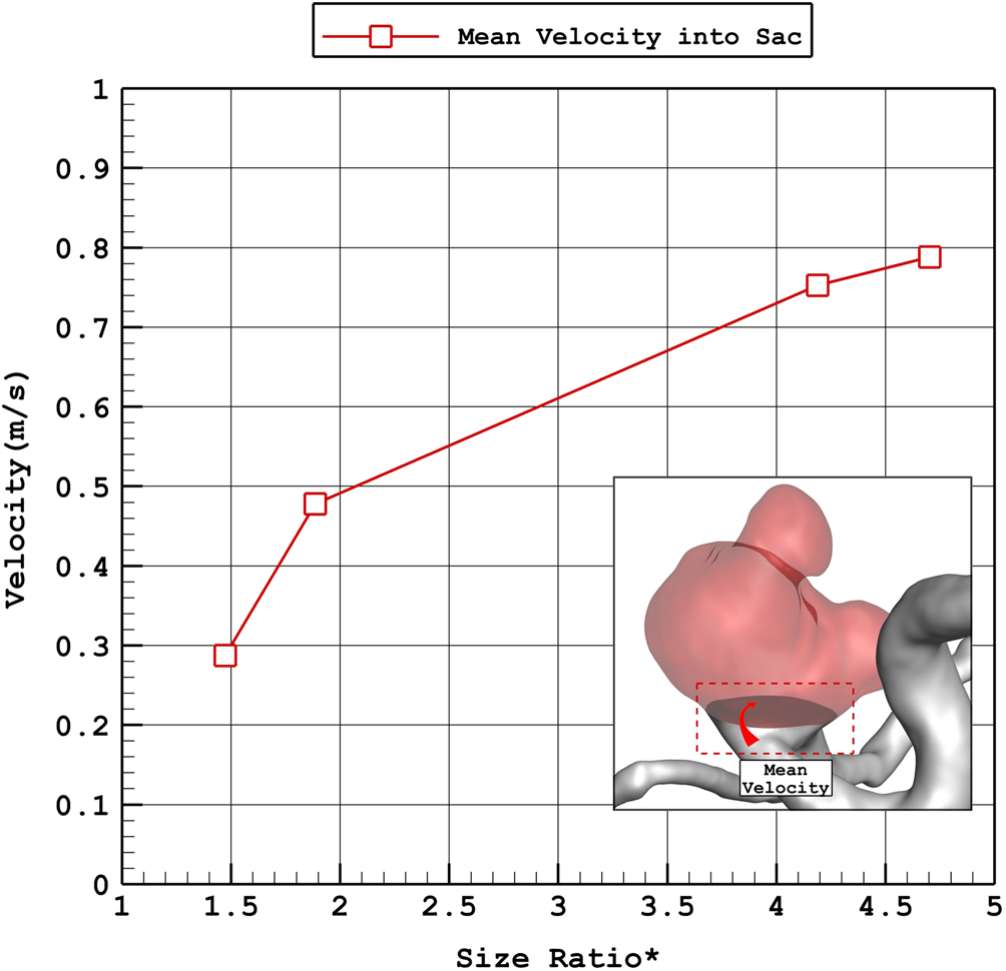
Variation of average velocity into the aneurysm Vs. size ratio* (peak systolic).

**Figure 14:**
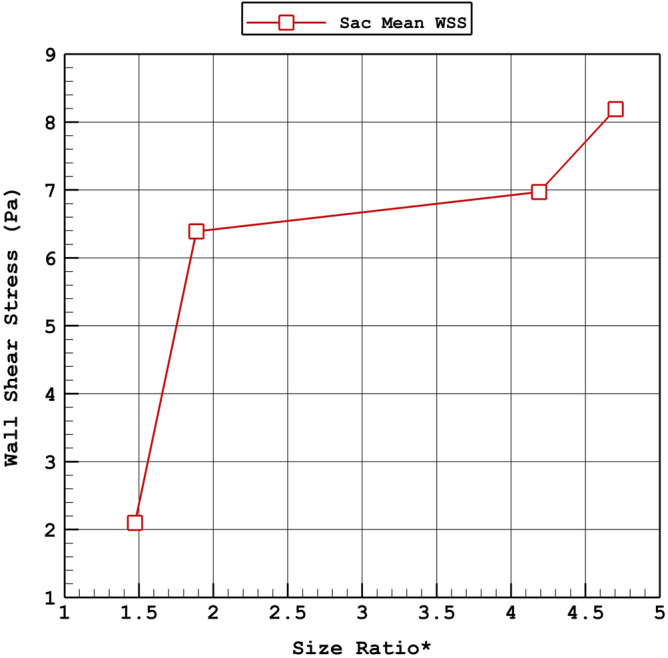
Variation of mean WSS Vs. size ratio* (peak systolic).

Figure 13 illustrates the influence of size ratio on the velocity of incoming blood into the sac section. The changes of size ratio from 1.5 to 4.5 increases the velocity about 270%. The effects of size ratio on the wall shear stress (Fig. 14) also indicate that this factor directly changes the wall shear stress. The most considerable raise in WSS is noticed from the changes of size ratio from 1.5 to 2.

**Figure 15:**
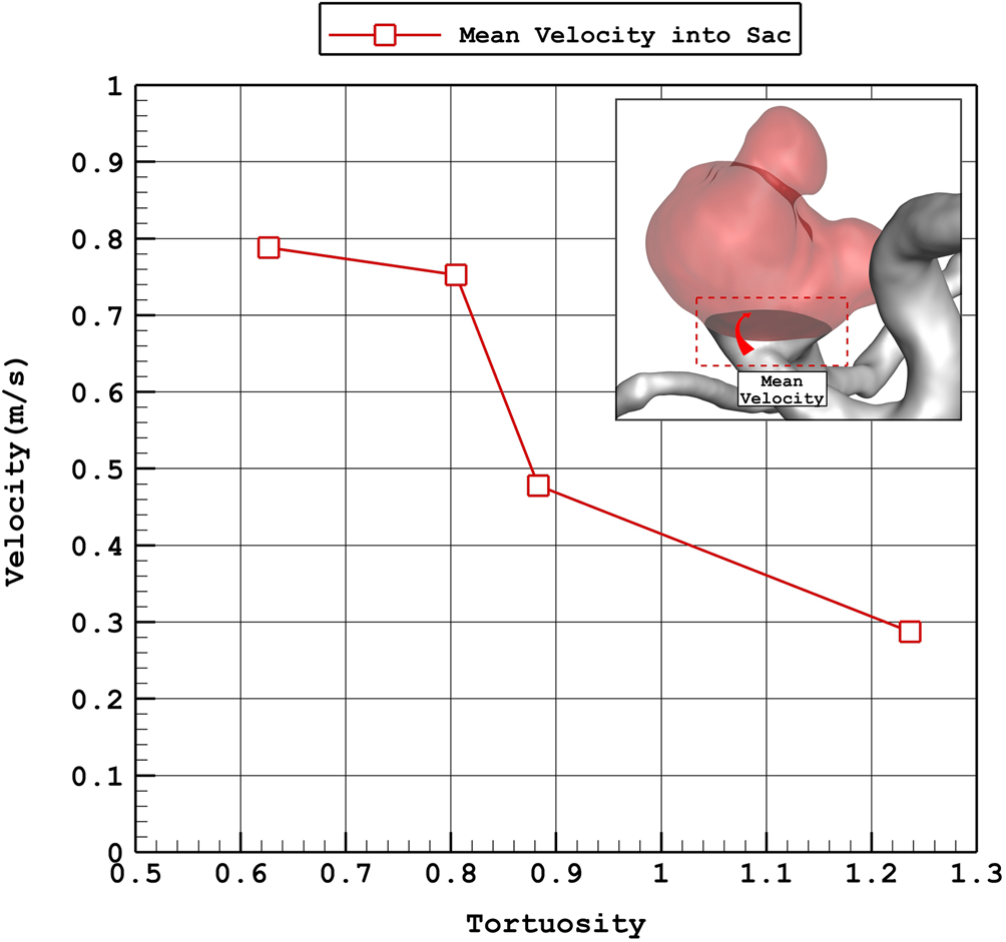
Variation of average velocity into the aneurysm vs. Tortuosity (peak systolic).

**Figure 16:**
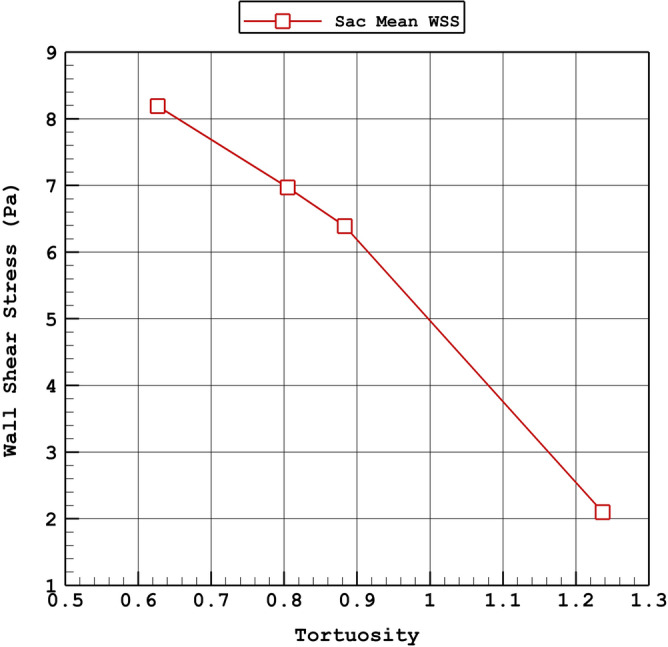
Variation of mean WSS vs. Tortuosity (peak systolic).

The influence of the tortuosity on the average incoming velocity and average WSS on sac wall is demonstrated in Figs. 15 and 16, respectively. As the tortuosity of the aneurysm is increased, velocity of the incoming blood into the sac is raised. Besides, mean wall shear stress is increased at peak systolic stage by increasing the tortuosity.

**Figure 17:**
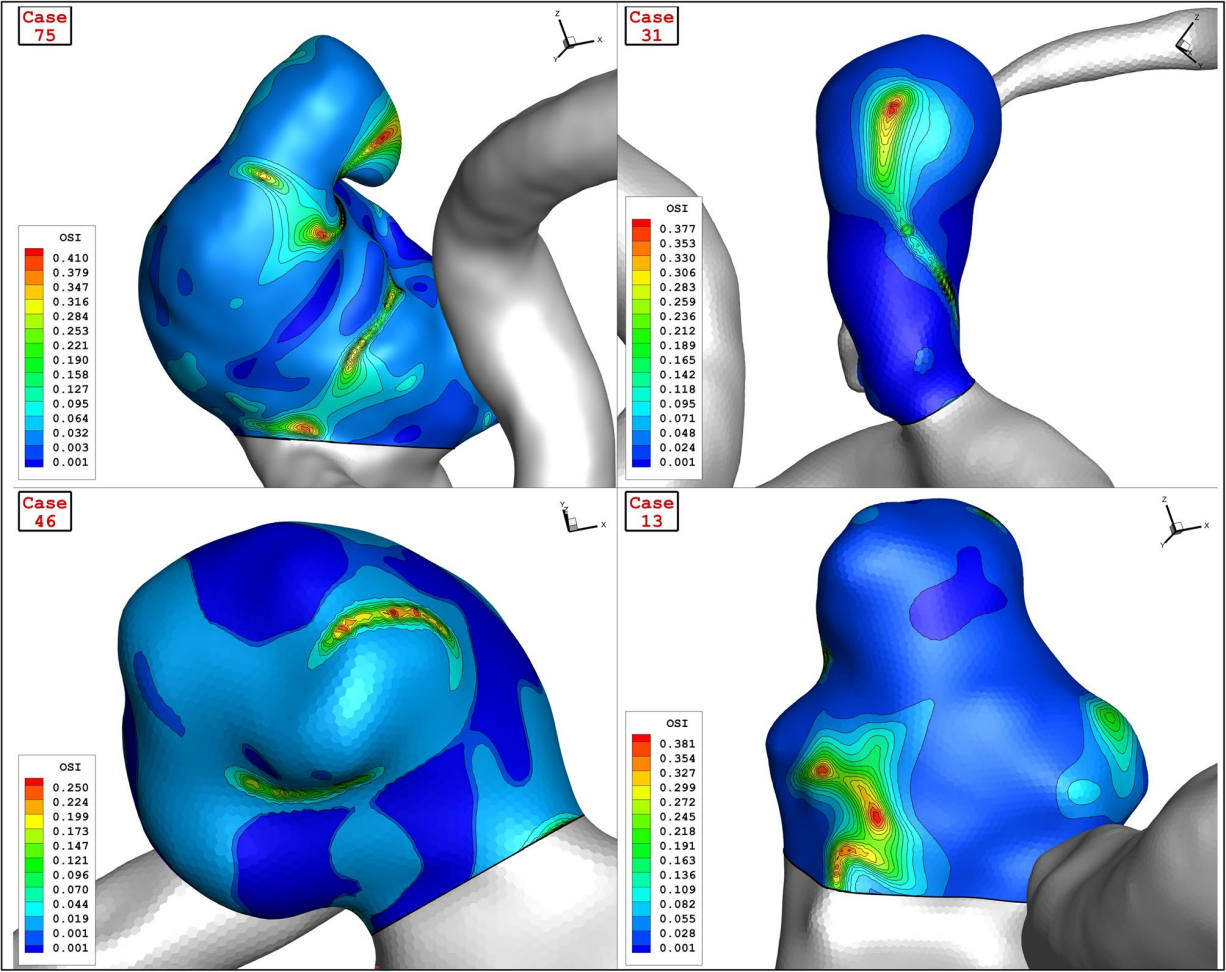
OSI contour of four selected cases (early diastolic).

**Figure 18:**
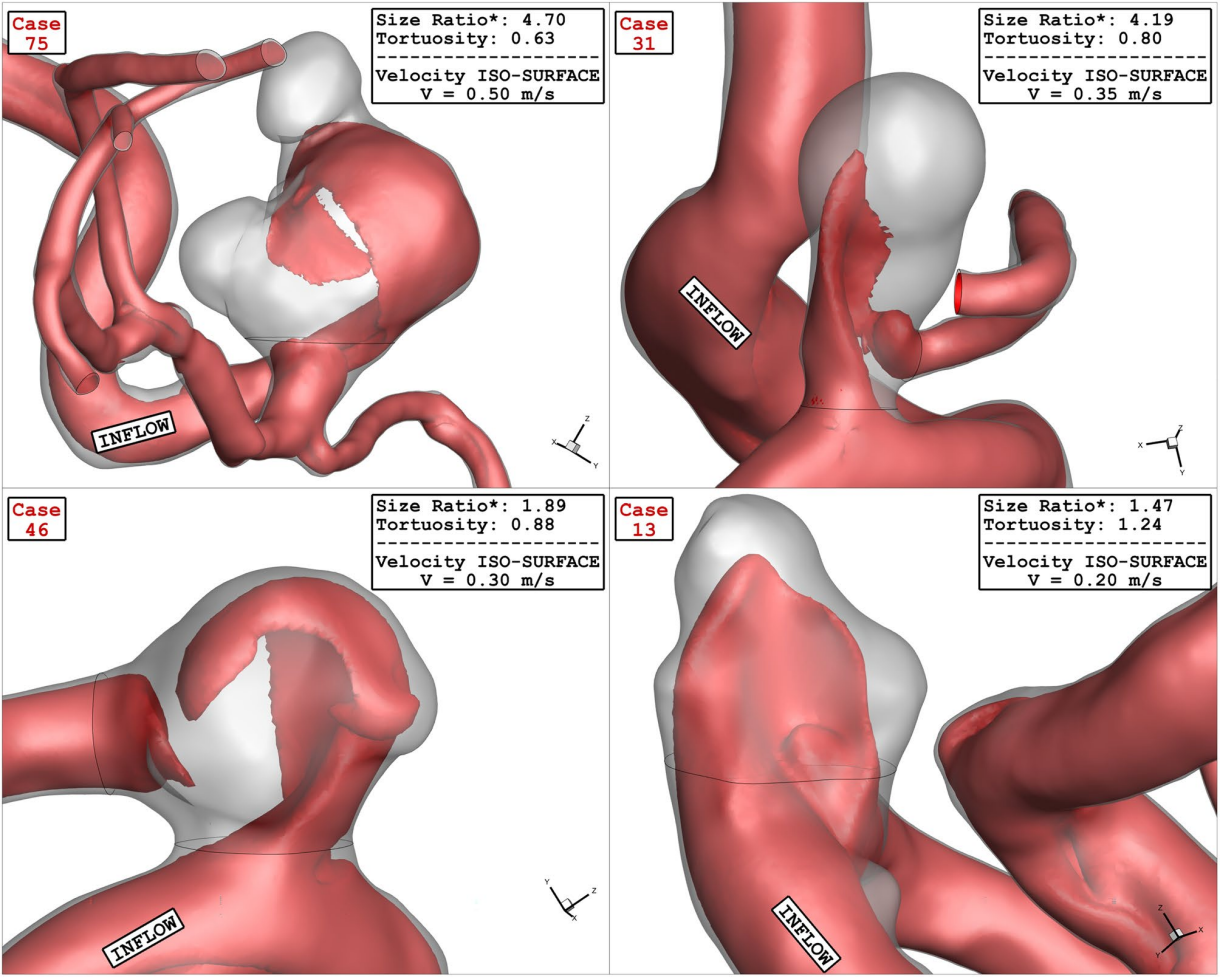
Velocity ISO-SURFACE of four selected cases (peak systolic).

**Figure 19:**
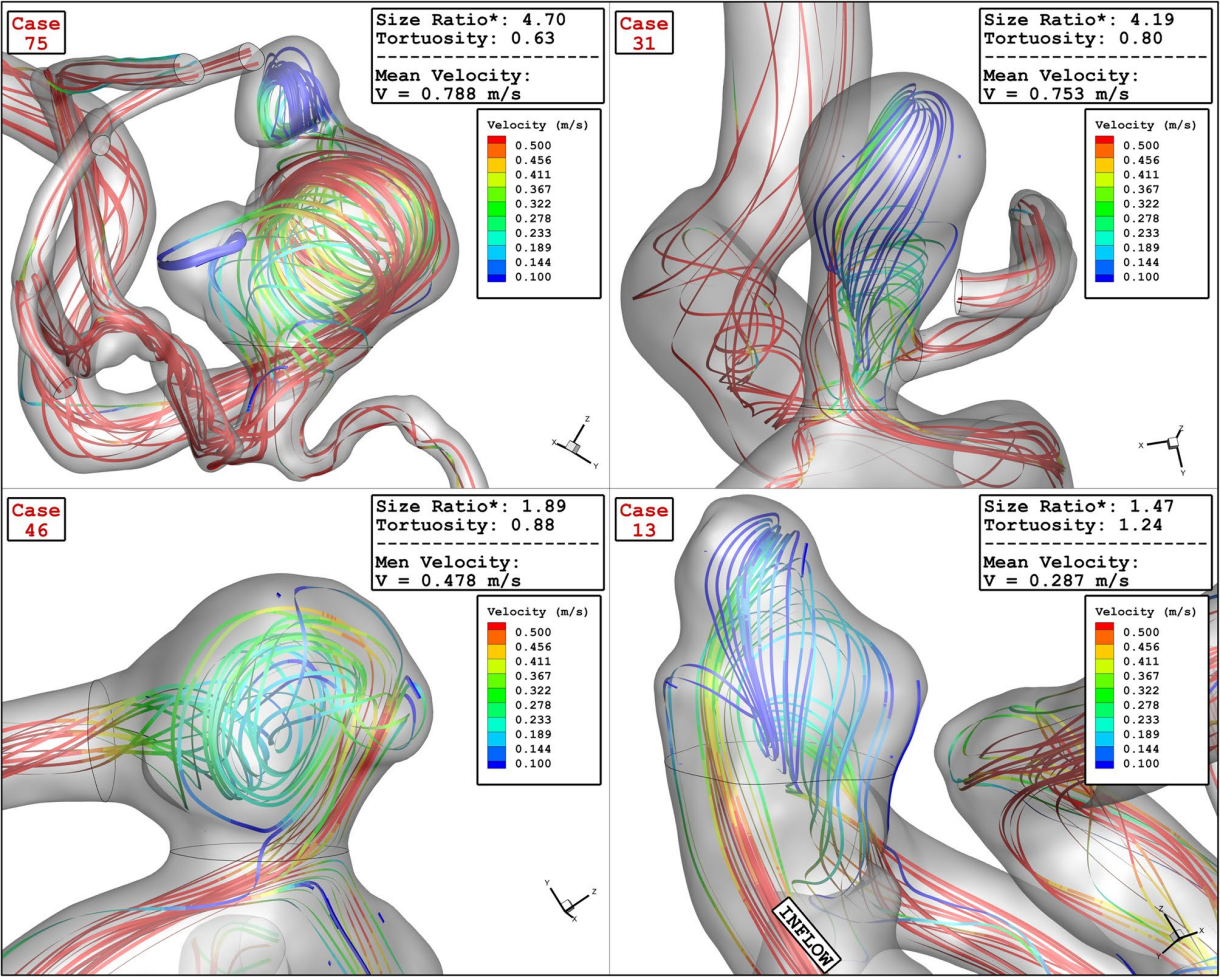
Velocity Streamlines of four selected cases (peak systolic).

Figure 17 displays the variation of OSI on sac wall for chosen aneurysms with different values of Ellipsoid Max Semi-Axis as significant index for the detection of the high-risk region. Comparison of the OSI values indicate that the OSI value is high near the dome region. Tor recognize the impact of blood hemodynamic, the blood iso-surface is displayed in Fig. 18. This contour shows how blood deformed the aneurysm shape at peak systolic stage. The blood stream inside the aneurysm and parent vessel in the different models with various values of Ellipsoid Max Semi-Axis is demonstrated in Fig. 19. The variation of the velocity on the blood stream is also demonstrated in this figure. The velocity gradient in sac region is more pronounced while the blood velocity is not changed in the parent vessel. The velocity of the blood after deflection from aneurysm wall decreases. In addition, the blood recirculation reduced the velocity of the blood which results in the velocity gradient in the aneurysm.

## Conclusion

This study investigates the effects of geometrical characteristics of cerebral aneurysms on the risk of rupture via hemodynamic analysis. Comprehensive statistical investigations are done to find connection between three geometrical features (Ellipsoid Max semi-axis, Size ratio and Tortuosity) and risk of aneurysm rupture over 30 different aneurysms. The statistical investigations confirm that rupture risk has direct connection with value of Ellipsoid Max semi-axis. Then, computational technique of CFD is applied to disclose how this factor influences on main hemodynamic characteristics of WSS, OSI and pressure on the sac wall. Critical regions prone to rupture is analyzed on four different aneurysms with diverse value of Ellipsoid Max semi-axis.

## Data Availability

All data generated or analysed during this study are included in this published article.
